# Spatial speech detection for binaural hearing aids using deep phoneme classifiers

**DOI:** 10.1051/aacus/2022013

**Published:** 2022-06-27

**Authors:** Hendrik Kayser, Hynek Hermansky, Bernd T. Meyer

**Affiliations:** 1 Auditory Signal Processing & Hearing Devices, Carl von Ossietzky University, 26111 Oldenburg, Germany; 2 Cluster of Excellence “Hearing4all”; 3 Center for Language and Speech Processing, Johns Hopkins University, Baltimore, MD 21218, USA; 4 Communication Acoustics, Carl von Ossietzky University, 26111 Oldenburg, Germany

**Keywords:** Direction-of-arrival estimation, Automatic speech recognition, Deep neural network

## Abstract

Current hearing aids are limited with respect to speech-specific optimization for spatial sound sources to perform speech enhancement. In this study, we therefore propose an approach for spatial detection of speech based on sound source localization and blind optimization of speech enhancement for binaural hearing aids. We have combined an estimator for the direction of arrival (DOA), featuring high spatial resolution but no specialization to speech, with a measure of speech quality with low spatial resolution obtained after directional filtering. The DOA estimator provides spatial sound source probability in the frontal horizontal plane. The measure of speech quality is based on phoneme representations obtained from a deep neural network, which is part of a hybrid automatic speech recognition (ASR) system. Three ASR-based speech quality measures (ASQM) are explored: entropy, mean temporal distance (M-Measure), matched phoneme (MaP) filtering. We tested the approach in four acoustic scenes with one speaker and either a localized or a diffuse noise source at various signal-to-noise ratios (SNR) in anechoic or reverberant conditions. The effects of incorrect spatial filtering and noise were analyzed. We show that two of the three ASQMs (M-Measure, MaP filtering) are suited to reliably identify the speech target in different conditions. The system is not adapted to the environment and does not require a-priori information about the acoustic scene or a reference signal to estimate the quality of the enhanced speech signal. Nevertheless, our approach performs well in all acoustic scenes tested and varying SNRs and reliably detects incorrect spatial filtering angles.

## 1 Introduction

A typical signal processing chain in a hearing aid includes speech enhancement followed by amplification and dynamic range compression for hearing loss compensation to alleviate recruitment effects, as well as feedback suppression to allow sufficiently high amplification. Audio processing conducted in the speech enhancement stage plays a crucial role in improving speech intelligibility, especially in noisy environments. The state-of-the-art approach for speech enhancement is to use deep machine learning and recurrent neural networks, specifically long short-term memory (LSTM) networks, which are often implemented as end-to-end systems [1]. These have also been considered as small-footprint LSTM systems which are compatible with hearing-aid hardware with good improvements in terms of the signal-to-distortion ratio [2], and are also efficient for the task of separating signals of multiple speakers [3, 4]. However, end-to-end systems are often trained as domain-specific systems [5], and even subtle changes in the signal processing properties requires a complete re-training of the neural network.

In this study, we therefore explore an extended, modular approach that exploits the potential of deep learning for hearing-aid signal processing and provides feedback about the obtained speech quality. We explore estimates of the spatial location of acoustic sources obtained from a direction-of-arrival (DOA) estimation algorithm, classical multi-channel beamforming in hearing aids, and speech quality measures based on deep learning. The DOA algorithm is based on support vector machines (SVMs) that estimate the DOA probability over time for azimuth angles of the frontal hemisphere relative to the listener [6]. A crucial aspect of the speech quality measures is that a reference signal, which is unavailable in real-world scenarios, is not required.

**Figure 1.**
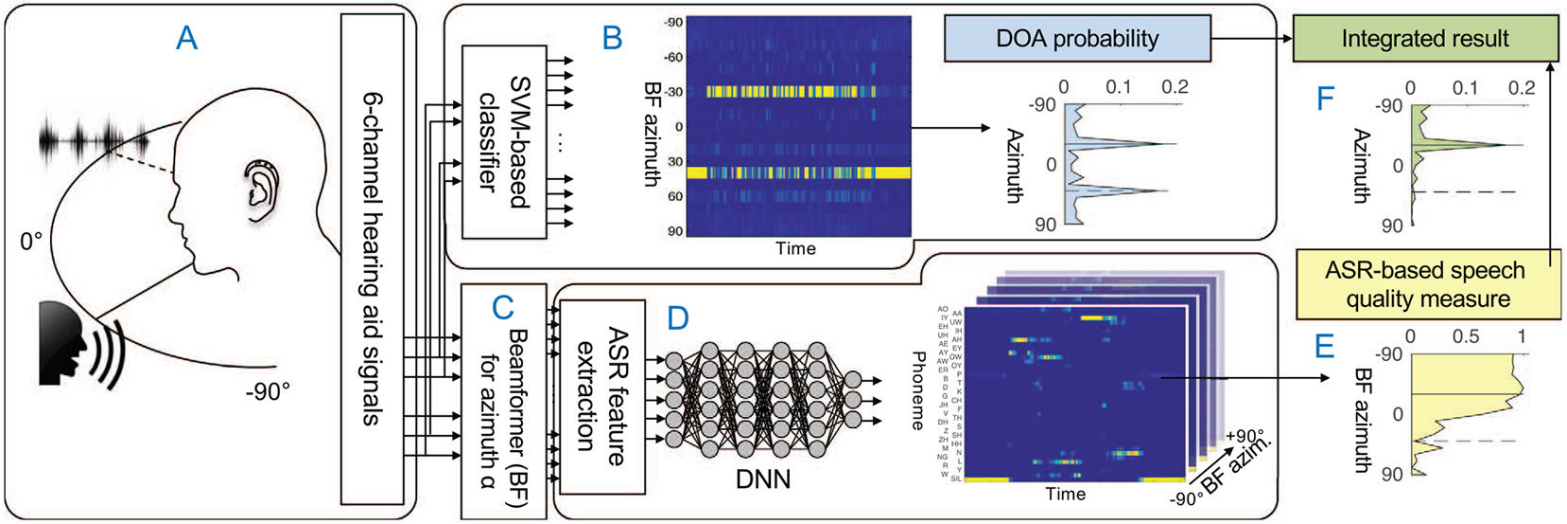
Illustration of the proposed system: (A, Sect. 2.1) A virtual acoustic scene (speaker at −30° and either a spatially diffuse noise or localized noise source at 40°) is captured with a 6-channel behind-the-ear hearing aid. (B, Sect. 2.2) Four channels are used to extract features for sound source localization fed to an SVM-based classifier. This results in a probability for direction of arrival of localized sound sources in the acoustic scene over time, which are averaged over time. (C, Sect. 2.3) A six-channel beamformer is used for spatial signal enhancement. (D, Sect. 2.4) ASR features are extracted from the beamformer signals and used as input to a DNN trained on speech data. This results in phoneme probabilities over time for each beamforming direction. (E, Sect. 2.5) An ASR-based speech quality measure is applied to these probabilities. (F, Sect. 2.6) By integrating information from two processing streams, the speaker at −30° (solid line) is clearly separated from the localized noise (dashed line).

Our aim is to combine these components to obtain a single-ended, speech-sensitive and spatially-aware algorithm that has the potential to optimize hearing aid parameters in realistic acoustic scenes. The proposed system combines two parallel processing paths as illustrated Figure 1: The first path exhibits a high spatial selectivity, but insensitivity to speech using the probabilistic DOA component (Fig. 1A); the second path has a low spatial selectivity, but high sensitivity to speech through beamforming and the speech quality measures (Fig. 1C-E).

The adaptive beamforming component processes multi-channel hearing aid signals, which often provides a substantial benefit for hearing aid users ([7, 8] and references therein). An adaptive procedure can exploit the spatial location of a sound source but it requires estimates such as the speaker’s position (assuming that hearing-aid users would like to attend a speaker in an acoustic scene) and noise statistics [9-11]. Incorrect parameter estimates for DOA or classical beamforming introduce artifacts in the output signal which potentially decrease speech quality and intelligibility [12]. Further, an adaptive beamforming approach by itself does not provide an implicit feedback regarding the speech quality that is delivered by the enhancement stage and it is therefore unclear if it performs well in a given acoustic scene. Such feedback is incorporated in our case by estimating the speech quality of the beamformed signals. To this end, these signals are used as input to a deep neural network (DNN) that is trained as phoneme classifier as part of a hybrid automatic speech recognition (ASR) system. The resulting phoneme probabilities are used to estimate the speech quality of the signals. We refer to the quality estimates as ASR-based speech quality measures (ASQM), which have been recently proposed in the context of speech technology, and which are described in the following: The DNN produces a representation of phonemes at its output – phoneme posterior probabilities over time (*posteriorgram*). The ASQMs exploit the fact that noisy or otherwise corrupted speech often results in phoneme activations that are smeared over time and become less sparse. Three ASQMs are considered in the current study: (i) The mean temporal distance or M-Measure, which was originally applied for predicting error rates of ASR systems [13] and which implicitly takes into account phoneme duration and co-articulation effects. The M-Measure was shown to accurately predict phoneme errors, and was later applied to select a feature stream in a multi-stream ASR system, which resulted in accuracies close to oracle performance (i.e., selection of the optimal stream) [14]. (ii) Similarly, an ASQM based on matched filtering of phoneme activations (MaP) was introduced in [15] and later applied to predict word error rates (WER) of ASR systems in unknown noise conditions [16]. Both M-Measure and MaP were explored in the context of hearing aid signals [17], and were shown to be informative for hearing aid parameters (such as settings for spatial filtering). (iii) Entropy of phoneme vectors, averaged over time, is explored since it should capture the sparseness of posteriorgram activations, i.e., the clear, distinct phoneme activations associated with clean speech should result in sparse posteriorgrams (and a low average entropy).

In a related work, the ASQMs that are utilized in the current study were also considered in spatial scenes; however, the main focus was the prediction of word error rates of ASR in spatial scenes and the hardware requirements [18], and DOA estimation was not used in this case. One of the main outcomes was that smaller neural networks for phoneme classification borrowed from an ASR system combined with the M-Measure could be run on hearing aid co-processors in real-time. To analyze the properties of the three ASQMs, we first quantify their relation to ASR word error rates (WER). This is motivated by the fact that ASR was shown to resemble human perception and is suited as a single-ended model for speech quality [19, 20]; also, we assume that the parameters that are systematically varied in our study (such as the SNR) should similarly affect ASR error rates and quality perception and hence an analysis of WERs could provide insight of how well the ASQMs generalize across different acoustic scenes. In order to evaluate the combined system, the performance of identifying a speech source location in different noisy environments is measured. To this end, different acoustic scenes (anechoic and reverberant, diffuse and a localized noise masker in a wide range of SNRs) are explored.

## 2 Methods

The basic concept of the proposed system (as illustrated in Fig. 1) is as follows: The audio signal that is captured by a six-channel behind-the-ear (BTE) hearing aid device (Sect. 2.1) is processed in parallel on two paths: a sound source localization algorithm (Sect. 2.2) and, after applying a beamformer (Sect. 2.3), a phoneme recognition module, which is borrowed from an ASR system but omits the decoding step through a hidden Markov model (Sect. 2.4). The information extracted in each path, i.e., spatial probability of sound sources present in the acoustic scene (DOA probability in Sect. 2.2) and ASR-based speech quality measure (Sect. 2.5) computed on the output of the speech recognition system, the phoneme posteriorgrams (from Sect. 2.4), were combined to a joint measure of direction-dependent speech quality (Sect. 2.6). Detailed descriptions of the different processing blocks are given in the following.

### 2.1 Speech signals, acoustic scene, and microphone setup

**Figure 2.**
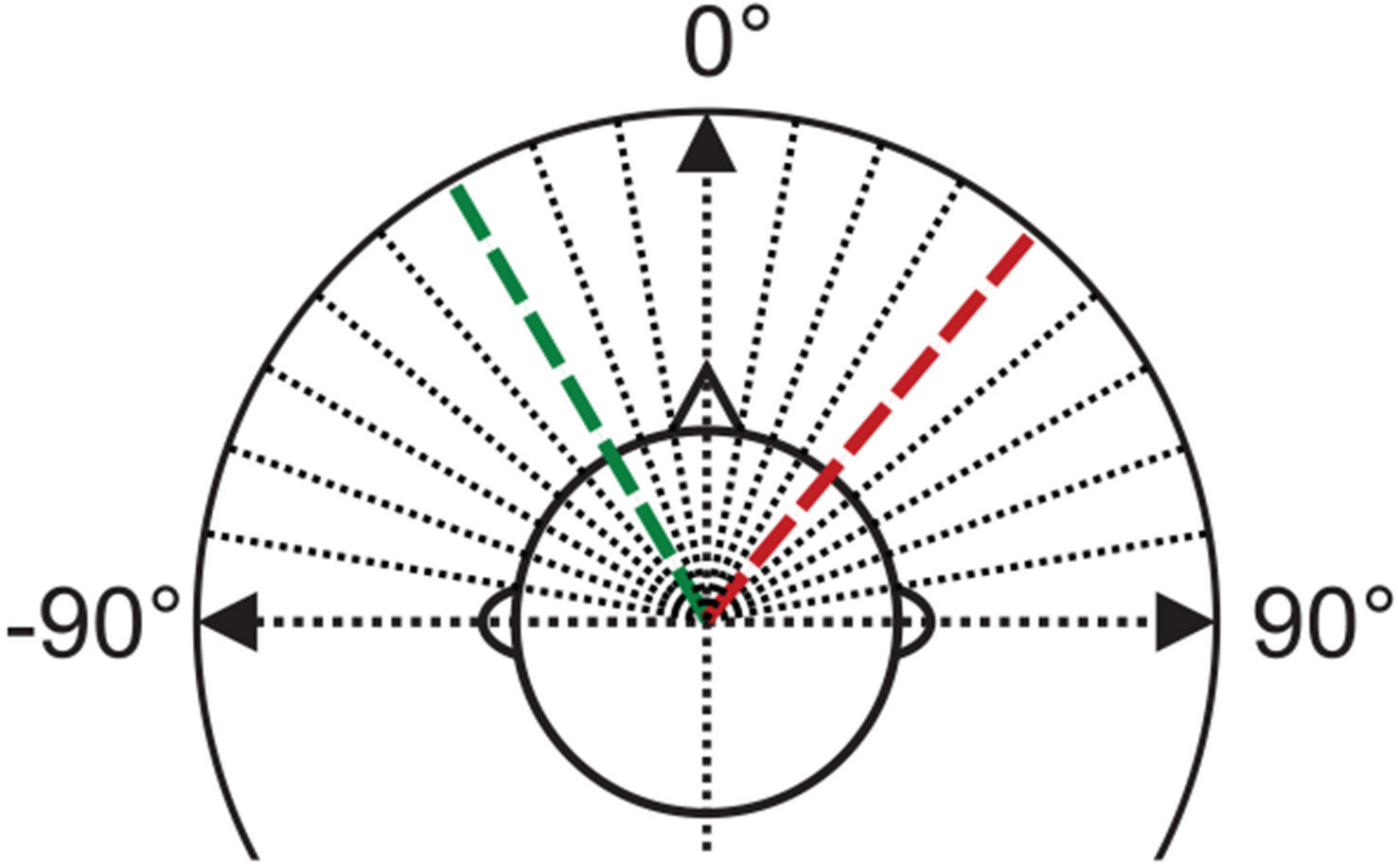
Coordinate system used in this study. The dotted (and colored dashed) lines depict the centers of the directional sectors as used for the beamforming and in the evaluation of the localization performance. The green line denotes the target speech direction, the red line indicates the noise position in case of a localized interferer.

Hundred utterances randomly chosen from the standard eval92 clean test set as proposed for Aurora 4 [21] were used as a testing base set. Aurora 4 contains read sentences from the Wall Street Journal, produced by native US English speakers. Spatial acoustic scenes were simulated by first convolving each utterance with recorded head-related impulse responses (HRIRs) obtained from a behind-the-ear hearing aid with three microphones on the left and right side of a dummy head, respectively [22]. The coordinate system is defined as depicted in Figure 2. HRIRs from an anechoic and an office room were used to simulate a speaker at an azimuth angle of −30° (green dashed line in Fig. 2), i.e., on the left-hand side of the virtual listener. Two types of noise were added to each utterance: Diffuse stationary noise, which was obtained by superimposing speech-shaped, stationary noise signals [23] from 19 directions, as well as a weakly modulated, localized noise signal (vacuum cleaning noise obtained from the BBC Sound Effects Library [24]) placed at 40° (red dashed line in Fig. 2). The SNRs ranged from −10 to +20 dB in steps of 5 dB. In total, 2800 spatial utterances were generated (100 utterances × 2 rooms × 2 noise types × 7 SNRs).

### 2.2 Spatial probability map estimation

**Figure 3.**
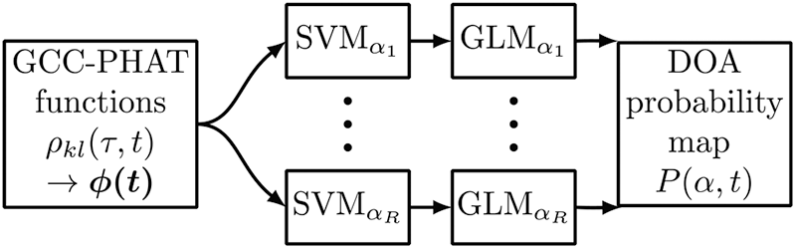
Flowchart of the direction-of-arrival (DOA) estimator. The signals captured by the microphone array are cross-correlated in short temporal segments *t* using the generalized crosscorrelation (GCC) with phase transform (PHAT). The resulting GCC-PHAT functions *p_kl_*(*τ, t*) from different microphone pairs (*k, l*) are combined based on a physically plausible range of delay *τ* to the feature vector *ϕ(t)*. Each *ϕ(t)* is classified by a set of R support vector machine (SVM) models trained for different DOA angles *α*_1_…*α*_R_. The resulting decision values are converted into the source presence probability estimate *P*(*α, t*) of direction α via a trained generalized linear model (GLM).

A spatial probability map represents the probability *P*(*α, t*) of the DOA of a localized sound source for each considered angle *α* at time frame *t* of the short-term Fourier Transform (STFT) *X*(*ω, t*) of the input signal. In this study, *P*(*α, t*) was estimated by using a classification approach to sound source localization [6]. Its structure is outlined in Figure 3. The input features of the system are generalized cross-correlation (GCC [25]) functions with phase transform (PHAT), given between microphone channels *k* and *l* by

(1) $$\rho_{{}_{kl}}(\tau ,t)=\frac{1}{\Omega }\sum_{\omega =0}^{\Omega }\Psi (\omega ,t)\cdot X_{k}(\omega ,t)X_{l}^{*}(\omega ,t)\cdot e^{i\omega \tau },$$

where *ω* is the angular frequency, *τ* is the delay and

(2) $$\Psi (\omega ,t)=\frac{1}{|X_{k}(\omega ,t)X_{l}^{*}(\omega ,t)|}$$

the PHAT frequency weighting. These GCC-PHAT features were extracted from short segments of the multi-channel input signal for physically plausible ranges of *τ* between all possible combinations input channel pairs and concatenated to the feature vectors *ϕ(**t**). ϕ(**t**)* was passed through a linear support vector machine (SVM [26]). The SVM system (LIBLINEAR [27]) was previously trained on the hearing aid microphone setup described above for a single speech source in a spatially diffuse noise field in anechoic conditions. Speech material was taken from the TIMIT speech corpus [28] and the spatially diffuse noise field was generated by convolution of a speech-shaped ICRA noise signal [29] with HRIRs from all azimuth directions in the horizontal plane and subsequent superposition of all resulting signals. For the training of the SVM classifier, randomly chosen speech samples were convolved with the same anechoic HRIRs that were used for generating the anechoic test scenario and mixed with the noise signal at different SNRs: −15 dB, 0 dB, and 15 dB. In summary, the training data for the localizer used in this study was based on the same anechoic HRIRs to capture the sensor geometry, but there was no match between the speech and noise signals used in training and test.

This data-driven, training-based sound source localization approach does not require to compute a precise acoustic propagation model and yields some robustness and also generalization capabilities to unknown environments.

The presence of a source was inferred by

(3) D(α,t)=〈w(α),ϕ(t)〉+b(α),

where ⟨·,·⟩ denotes the scalar product, ***w**(α)* is the learned weighting vector to discriminate direction *α* against all other directions taken into account in the training data set, *b*(*α*) is a bias term and *D*(*α,t*) the resulting decision value. To obtain a probabilistic statement about source presence, each *D*(*α, t*) was subsequently mapped to the probability estimate *P*(*α, t*) using a generalized linear model (GLM) with parameters *β*_1_ and *β*_2_

(4) $$P(\alpha ,t)=\frac{1}{1+e^{-(\beta_{1}+\beta_{2}D(\alpha ,t))}},$$

which was fitted to the training data set by using *D*(*α, t*) together with the ground truth class labels available from the SVM training data. The output of this step is the spatial probability map *P*(*α, t*) that contains the estimated distribution of sound source presence probability over *α* at each time frame t as depicted in Figure 1B.

### 2.3 Beamforming

Beamforming was conducted in the frequency domain by multiplying the STFT of the six-channel input signal **X**(*ω, t*) with a spatial filter vector **w**(*α, ω*) yielding the output *Y*(*ω, t*):

(5) $$Y(\omega ,t)=w^{H}(\alpha ,\omega )\;X(\omega ,t)$$

with the frequency-dependent spatial projection operator **w**(*α, ω*). Minimum-variance distortionless-response (MVDR [30]) beamforming is applied, yielding

(6) $$w(\alpha ,\omega )=\frac{R^{-1}(\omega )\;d(\alpha ,\omega )}{d^{H}(\alpha ,\omega )\;R^{-1}(\omega )\;d(\alpha ,\omega )}$$

with the steering vector **d**(*α, ω*) and the noise covariance matrix **R**(*ω*). For **R**(ω) a head-related isotropic noise field [31] computed from anechoic head-related transfer functions was chosen. This way, the general assumption of a spatially diffuse noise field which does not contain specific room information is realized.

The room-independent properties of the receiver, i.e., the characteristics of the head-mounted microphone array are taken into account. **d**(*α, ω*) was computed from the anechoic head-related transfer functions according to the direction *α*. Each of the 2800 multi-channel utterances was processed with spatial signal enhancement with steering vectors directed at angles *α* ranging from −90° ≤ *α* ≤ +90° with a resolution of 10°, which resulted in a total of 53,200 (2800 × 19 directions) single-channel utterances for the test set. This data was later used to explore the effect of spatial enhancement at different azimuth angles.

### 2.4 ASR system and phoneme posteriorgrams

The ASR system exploited for this study uses mel-filterbank features as input and covers an acoustic model that maps features to phoneme probabilities (i.e., the DNN), as well as a Hidden-Markov-Model (HMM) that decodes frame-wise observations to obtain a transcript of the utterance. Note that the HMM is not required for a later application of the model in the context of hearing aids, which relies on the output of the DNN alone. The ASR was implemented in the open source software [32] following the standard procedure for the multi-condition Aurora 4 task [33], which contains read speech (news from Wall Street Journal) recorded from 83 native US English speakers (7137 utterances) for a total of 14 h in the training set. Training and test sets contain data recorded from different speakers. The test utterances (as described in Sect. 2.1) also contain read speech from the same newspaper. Multi-condition training used a mix of clean and noisy utterances with additive noise in the range from 10 to 20 dB SNR. The following noise types were covered in the training set (none of which is used in the test set): airport, babble, car, street, and restaurant. Clean training was also considered, but resulted in ASR systems with inferior robustness to noise and therefore high WER even in moderate amounts of noise, and was therefore not further used.

We first extracted mel-filterbank features by using 25 ms Hamming windows with a 10 ms shift and subsequent frequency analysis with 512 frequency bins. These are grouped with 40 triangular filters that were equidistant on a mel scale, resulting in higher resolution of lower frequencies. By taking the logarithm of the magnitude of the filter outputs, one feature vector per frame was obtained. To provide temporal context, the 40-dimensional vector was concatenated with five preceding and five following frames, creating 440-dimensional input vectors for the DNN. The DNN used six hidden layers, 2048 units per layer, and an additional softmax output layer. It was pre-trained as a restricted Boltzmann machine using contrastive divergence and supervised fine-tuning with context-dependent triphone targets via cross entropy. The output activations of the DNN correspond to phoneme posterior probabilities for each time frame with a shift of 10 ms. In total, 2026 output neurons that correspond to context-dependent triphones were used. All phones were modeled with three HMM states, and silence is modeled using five states. After DNN training, discriminant sequence training using Minimum Bayes-Risk was performed, which is a standard procedure of the kaldi recipe (Aurora 4) employed here since it provides increased performance for medium and large vocabulary ASR. For each time frame, the DNN produces one 2026-dimensional output frame of triphone activations as stated above. For the purpose of speech quality prediction, this output is grouped as described in the next subsection.

### 2.5 ASR-based speech quality measures

To obtain a speech quality measure based on phoneme posteriorgrams, we first grouped all triphones that belong to the same phone. This reduced the number of activations from approximately 2000 to 40, which made the following steps computationally efficient, while having no significant impact on performance [17]. To quantify the quality of posteriorgrams, we explored three different approaches that are described in the following.

#### 2.5.1 Mean temporal distance measure – M-Measure (MM):

The mean temporal distance measure (M-Measure) was used to quantify degradation of the posteriorgram and therefore serves as a speech quality measure. It was first proposed as the predictor for phoneme error rates [13] and later applied for selecting the most reliable streams in multi-stream ASR [14]. It is motivated by the temporal smearing of phoneme posteriorgram activations that are often observed for noisy speech representations. The M-Measure is based on the average difference of two phoneme posterior vectors **P***_ph_* (*t* – Δ*t*) and **P**_*ph*_ (*t*) with a temporal distance Δ*t*, and is given by

(7) $$M(\Delta t)=\frac{1}{T-\Delta t}\sum_{t=\Delta t}^{T}D\left(P_{ph}(t-\Delta t),\;P_{ph}(t)\right),$$

where *T* is the duration of the analyzed posteriorgram which is equal to the length of one utterance in this study. The Kullback–Leibler divergence was used as the distance measure *D*. The original M-Measure considered time delays from 10 ms to 50 ms in steps of 10 ms, and from 50 ms to 800 ms in steps of 50 ms. On average, the M-Measure curves increase from 0 ms to 200 ms (since it becomes more and more unlikely that the current phoneme stays “activated”), and saturate after this (since phoneme durations and coarticulation effects over 200 ms are atypical). For our approach, we relied on this saturated part of the curve and used Δ*t* values from 50 ms to 800 ms, which were averaged over the length of one utterance to obtain one scalar value per test item. We used this scalar value to test the hypothesis that lower average values of the M-Measure indicate a degradation of the phoneme posteriorgrams.

#### 2.5.2 Matched Phoneme (MaP) filters

**Figure 4.**
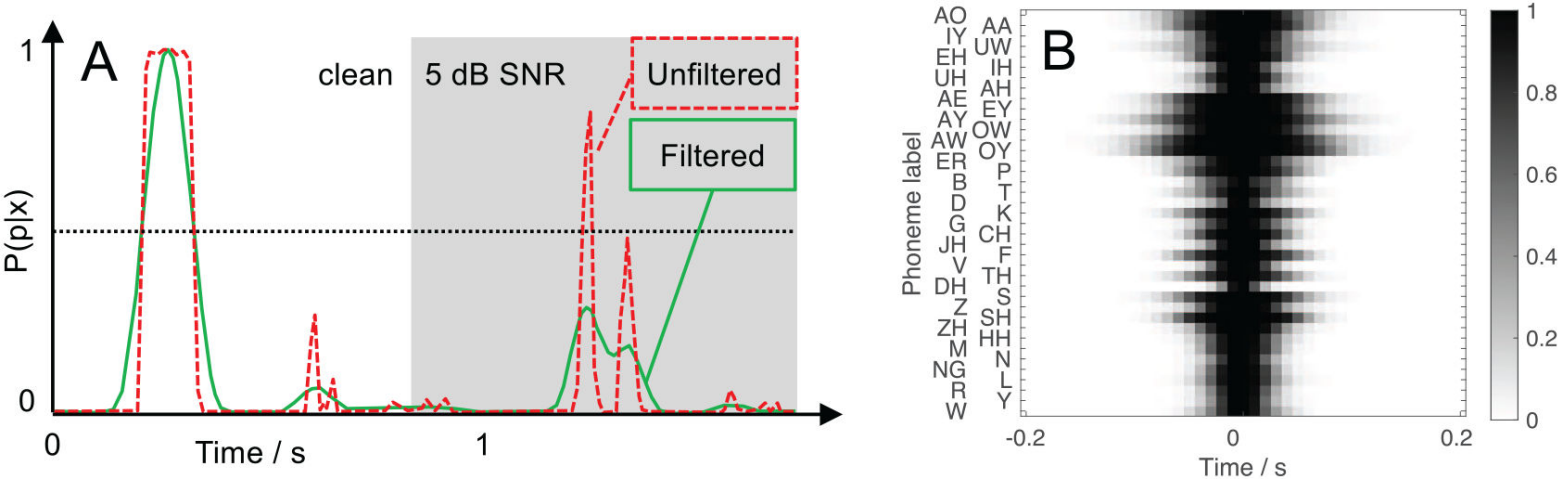
Panel A: Illustration of matched filtering: The clean activation of the phoneme /k/ (dashed red curve, clean condition on the left side) has a high match with the learned filter. Hence, the filter output (the convolution between filter and the activation) is high (solid green curve, filtered). The same phoneme in noise (5 dB SNR, gray background on the right side) results in an atypical activation (dashed red curve, right side), resulting in a filter output with smaller values. Panel B: Matched filters learned from the TIMIT speech database (high activation values (probabilities) are shown in black).

As a second measure of speech quality, matched filtering of phoneme activations was explored. This method is based on the observation that signal degradations often result in inconsistent activations of phonemes, which could result in a poor match between activations (filters) learned from clean speech data and the activation of the observed signal. The first step of this procedure is to calculate the convolution

(8) $$M_{p}[k]=h_{p}[k]*a_{p}[k]$$

with the learned filter *h_p_*, the phoneme activation *a_p_* for each phoneme with index *p*, and *k*, the index of the time frame. We except high filter output values for a good match between the optimal filter shape and the actual activation obtained from the DNN and lower output values for degraded phoneme representations. An example is shown in Figure 4A: The clean activation of the phoneme /k/ resembles a rectangle (dashed line, left panel), while the identical phoneme in noise exhibits two peaks (right panel). In the example in Figure 4A, the normalized output from matched filtering is shown by the solid lines. Both the clean and noisy phoneme can be easily separated with a 0.5 threshold on the filter output (as suggested by [34]), which is in contrast to the unfiltered case. In our study, we used a filter set learned from clean TIMIT speech data and a threshold criterion of 0.5 as proposed in [16]. This set contained filters for each phoneme (illustrated in Fig. 4B), which are convolved with the monophone posteriorgram. Since above-threshold events are less likely to occur for degraded data, we used the number of above-threshold activations after filtering (which we refer to as phonetic events) as a scalar value for further analyses. In contrast to the previous measure, matched filtering specifically models phoneme-specific durations and can also account for asymmetric activations as long as these are contained in clean posteriorgrams.

#### 2.5.3 Inverse entropy (iEnt)

The inverse entropy was employed as a baseline speech quality measure since it was shown to provide reliable results as a confidence measure in ASR [35]. Further, the entropy of phoneme posteriorgrams was found to relate to degradation of spatially-filtered, noisy speech signals in a previous study [36]. The motivation behind using inverse entropy is that degraded speech should result in multiple class activations in the phoneme posteriorgram and therefore a low inverse entropy (in contrast to one clear peak among many classes, which corresponds to a high inverse entropy). It was calculated based on the average frame-wise entropy of the posteriorgram for a complete utterance:

(9) $$H=\frac{T}{\sum_{T}\sum_{ph=1}^{N}-\;P_{ph}(t)\log_{2}\left(P_{ph}(t)\right)},$$

where **P**_*ph*_(*t*) is the activation of the *ph*-th phone, *N* is the total number of phones, and *T* is the same as for Equation (7).

### 2.6 Combination of localization information and ASR-based speech quality measure

In the current experiment, the effect of spatial filtering on speech quality was assessed utterance-wise.

Aiming at a probabilistic combination of ASR-based speech quality measures (ASQM) and spatial probability, the ASQM was rescaled for each utterance and interpreted as a probability:

(10) $$P_{\text{ASQM}}(\alpha )=\frac{\text{ASQM}(\alpha )-\text{min}(\text{ASQM}(\alpha ))}{\max(\text{ASQM}(\alpha ))-\text{min}(\text{ASQM}(\alpha ))}.$$

The spatio-temporal source probability maps were averaged over the length *T* of one utterance resulting in one probability function for each utterance:

(11) $$P_{\text{DOA}}(\alpha )=\frac{1}{T}\sum_{t}P(\alpha ,t).$$

Both are combined to the joint spatial speech likelihood function *P_s_*(*α*):

(12) $$P_{s}^{\text{DOA}\times \text{ASQM}}(\alpha )=P_{\text{ASQM}}(\alpha )\cdot P_{\text{DOA}}(\alpha ).$$

*P*_s_(*α*) reflects the likelihood of good performance of the ASR system for a spatial signal enhancement steered toward direction *α*.

We hypothesize that a high *P*_s_(*α*) at the same time indicates successful application of the speech enhancement system and therefore predicts a benefit for a hearing aid user given the according setup of the system. In contrast, by simply steering the spatial filter in the direction of the most likely sound source the system may fail in presence of a dominating localized non-speech interferer.

In order to investigate this effect, we evaluated the system in terms of speech source localization performance in presence of a diffuse and a localized interfering noise source. Assuming that the maximum of *P_s_*(*α*) indicates the direction of arrival of a speech source, speech DOA estimation was conducted by

(13) $${\hat{\alpha }}_{s}^{\text{DOA}\times \text{ASQM}}=\underset{\alpha }{\text{arg}\;\max}P_{s}^{\text{DOA}\times \text{ASQM}}(\alpha ).$$

Alternatively, utterance-wise DOA estimation could be conducted on the two other probability estimates – the source DOA probability *P*_DOA_(*α*):

(14) $${\hat{\alpha }}_{s}^{\text{DOA}}=\underset{\alpha }{\text{arg}\;\max}P_{\text{DOA}}(\alpha )$$

and based on the purely speech-quality based *P*_ASQM_(*α*):

(15) $${\hat{\alpha }}_{s}^{\text{ASQM}}=\underset{\alpha }{\text{arg}\;\max}P_{\text{ASQM}}(\alpha ).$$

The hit rate is computed as

(16) Hit rate=100U∑u=1UΘu(α^s),Θu(αs)={1,α^s=αs0,otherwise}

with the true speech source position *α_s_* over the total number of utterances *U*. In that context *α_s_* refers to the directional sector in which the target source is located. The directional sectors are given by the beamforming direction *α* as defined in Section 2.3 with −90° ≤ *α* ≤ +90° with a resolution of 10°. This effectively yields a tolerance of ±5° around the true source position and a chance level of 1/19 ≈ 0.0526.

## 3 Results

### 3.1 Relation of ASR-based speech quality measures and word error rate

**Figure 5.**
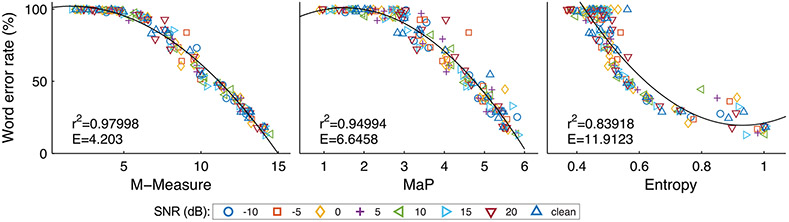
Word error rate over ASR-based speech quality measure (ASQM) values. Each data point corresponds to a specific noise type, SNR, beamforming angle, and environment (office or anechoic). Color/symbol denotes the SNR. The solid curve in each panel denotes a second-order polynomial fit to the data points between the according ASQM and word error rate. The correlation value r between fit and data and the goodness of the fit in terms of root mean square error *E* are given in the left bottom corner of each panel.

To gain some insight into the properties of the ASQMs, specifically how well they generalize across the different acoustic parameters in this study, we compared the values obtained for our three ASQMs with the WER obtained from an ASR system, i.e., the WER that is observed when running the full ASR model including the HMM for decoding. The scatter plots that compare ASQM value and WER for the same utterances (and conditions) are shown in Figure 5. Each data point corresponds to one of eight noise levels (from −10 dB to 20 dB SNR and clean), a specific noise type (diffuse or vacuum cleaner), beamforming azimuth *α*, and room type. We fitted a second order polynomial to the data points for each type of ASQM to reflect the strength of the relation between ASQM and WER. The resulting correlation and goodness of the fit were highest for M-Measure (*r* = 0.98, RMSE = 4.20), followed by MaP (*r* = 0.95, RMSE = 6.64) and entropy (*r* = 0.84, RMSE = 11.91). For the first two measures (MM and MaP) the variance was found to be much smaller than for entropy (iEnt).

### 3.2 Speech detection and localization

**Figure 6.**
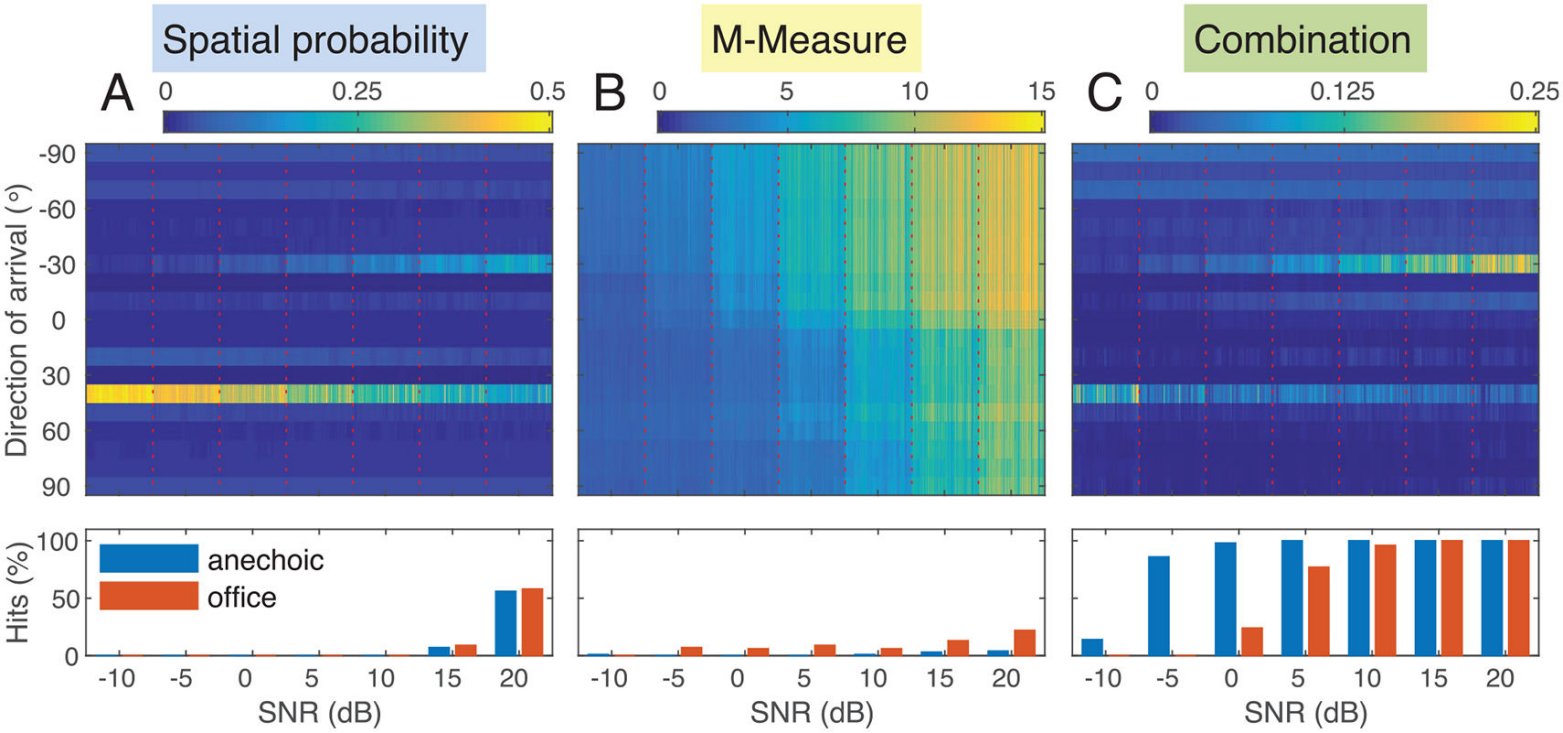
Results based on the M-Measure obtained for a single speech source with −30° DOA and a localized interferer (vacuum cleaner) with 40° DOA at varying SNR (−10 dB to 20 dB, abscissa). Top row: Results are shown for the office environment. Outcomes of stages B, E, and F in Figure 1. Left panel (A): DOA-dependent probability of sound source presence *P*_DOA_(*α*) (Eq. (11)). Center panel (B): M-Measure (MM, Eq. (7)) obtained from phoneme posteriorgram output of a deep neural network for speech recognition as a measure of direction-dependent speech quality. Right panel (C): Combination of spatial source probability and ASR-based speech quality measure $p_{s}^{\text{DOA}\times \text{ASQM}}(\alpha )$ (Eq. (12)) recovers the speech source location from the mixture with the dominant interferer. Bottom row: Speech location detection performance (hit rate) based the information presented above and using Equations A: (14), B: (15), and C: (13) in the anechoic (blue bars) and the office (red bars) environment.

The proposed system was evaluated for the three described ASQMs (M-Measure, Matched Phoneme filters and inverse entropy). Speaker location detection was conducted based on Equation (13). In Figure 6 probability maps obtained with the M-Measure in the office environment with one speaker and a localized noise source (vacuum cleaner) are shown in the top row. The performance in terms of hit rate, see Equation (16), is shown in the bottom panels for both environments. The data displayed is A: spatial source probability map *P*_DOA_(α) (top) and hit rates for the correct speaker localization based on the spatial probability (bottom) dependent on the SNR (see abscissa); B: DOA-dependent M-Measure and detection performance based on the speech quality measure only; C: combination of spatial probability and ASR-based speech quality measure $P_{s}^{\text{DOA}\times \text{ASQM}}(\alpha )$. For SNRs of 10 dB and below, the speech source at −30 is not selected as target for the spatial filter when only relying on the most likely DOA based on the probability of sound source presence (Panel A). This indicates that the DOA estimator may not reflect the direction of the target speech when other sound sources are present. Only at a very high SNR of 20 dB was the speech source selected in slightly more than half of the cases in both environments. The non-speech masker is selected in all other conditions.

The discrepancy between spatial and purely speech-related information is obvious in Panel B: The ASR-based speech quality measure shows highest values for the quadrant in which the speech source is located. Nevertheless, the resolution capability is not high enough to detect the DOA of the speech source accurately as reflected in the hit rates displayed below panel B in Figure 6. In combination (Panel C) high hit rates are achieved for −5 dB SNR (86%) or higher in the anechoic condition and for SNRs above 0 dB in the office room (≥ 77%).

In Tables 1 and 2, the speaker detection results are summarized for all possible cases: DOA probability only (Fig. 1B), the three different ASR-based speech quality measures ASQM (Fig. 1E) and the combination of DOA and ASQM: DOA × ASQM (Fig. 1F).

#### 3.2.1 Diffuse noise

Table 1 contains the results for the diffuse noise condition. In this case, good results are obtained based on DOA information alone with a 100% hit rate in all SNRs in the anechoic environment and in the office room (with the exception of −10 dB SNR with a 97% hit rate). The interesting aspect in the diffuse noise condition is the effect of incorporating the ASR-based speech quality measures, as in the intended application there is no feedback to the algorithm as to its success in identifying the correct localization. As shown in the three ASQM columns containing the results that were obtained with the three different ASR-based speech quality measures, the ASR-based speech quality measures follow different trends, particularly in the anechoic environment. Under anechoic condition, MM achieves high (up to 99%) hit rates at the low SNRs (−10 dB to 0 dB) which noticeably decreases towards high SNRs. A similar trend is observed in the office room, but with considerable smaller hit rate of 35% at the lowest SNRs. MaP does not reach high hit rates in both environments; the highest rates are obtained for medium SNRs. iEnt produces the lowest hit rates on average with a consistent improvement from low to high SNRs. Results for the combined measure DOA × ASQM show the interaction effects between the the DOA estimation approach and each of the ASQMs: The combination of the DOA estimation algorithm and MM has practically no effect on the speech localization performance, i.e., there is no significant difference in the hit rate compared to using DOA alone. This means that DOA estimation performance in diffuse noise is at or close to 100% (as expected), and remains unaffected when combining DOA with the M-Measure.

The picture for the other two measures is different: In combination with DOA, both have a detrimental effect on the localization performance. Thereby a slight effect is observed for MaP in the anechoic environment – small decrease of hit rates for low and high SNRs compared to DOA alone – which becomes more distinct in the office room. iEnt causes a significant drop in performance at low SNRs in both environments, while it has no influence on the results for SNRs >5 dB.

#### 3.2.2 Localized interferer

Table 2 shows results for a localized interfering noise (vacuum cleaner) at a DOA of 40° based on the same environments and SNR parameters as before.

Regarding its spectrum and temporal dynamics, the vacuum cleaner noise clearly dominates the scene, since it has high energy in a wide frequency range and is less modulated compared to the speech source. These characteristics are also reflected in Figure 6A: In the spatial probability maps, the 40° row appears to be the dominating source location over the whole SNR range in the office environment. This is confirmed by the *SP* (speech) and *VC* (vacuum cleaner) columns in Table 2. Up to an SNR of 10 dB, the vacuum cleaner corresponds to the most probable DOA in both rooms. At 15 dB SNR, the speech location is identified as the target DOA in less than 10% of the cases, and at 20 dB SNR, the hit rate increases to only 60%.

Using ASQM information only for detecting the localized speaker yields very low hit rates in case of MM and iEnt. MaP achieves hit rates up to 75% in the anechoic condition and 79% in the office environment, but no consistent behavior between rooms or with SNR is observable. The situation is different when DOA is combined with ASQM. This is especially notable for the combination with the M-Measure, which outperforms both DOA alone as well as M-Measure alone in all conditions (with the exception of −5 and −10 dB SNR in the office condition). In both environments the incorrect identification of the vacuum cleaner as target source can be resolved to a larger extent. Considering that in this scenario there are two candidates for the target location the chance level for a hit is 50%. The lowest SNR condition under which results are clearly above chance level are achieved is −5 dB in the anechoic environment (86%) and 5 dB in the office room (77%).

Although for column ASQM MaP outperforms the DOA × ASQM approaches in some cases most of these results are close the chance level and there is no consistent behavior related to the SNR observable. The performance of iEnt combined with DOA is comparable with the combination of MaP and DOA in the anechoic scenario and smaller for the office environment.

## 4 Discussion

### 4.1 ASR training data

The DNN used in this study was trained with the standard multi-condition training set proposed for the Aurora 4 framework [21], and it seems likely that the training material can be optimized for further improvement by selecting speech sources. ASR performance (and therefore implicitly the robustness of DNN outputs) could certainly be improved by exposing the model to beamforming artifacts, which are not covered by the current training set. The same is true for ASR in reverberated environments, which should benefit from including speech convolved with room impulse responses in the training set. However, this increase robustness is not desired in case of the current study. A high sensitivity to beamforming artifacts is required to capture the degradation of speech due to suboptimal spatial filtering parameters in the context of hearing devices. Reverberation can have a detrimental effect on perceived speech quality, which should also be reflected by our system. An ASR system training that does not take into account such signal disruptions would cause the resulting ASQM being negatively impacted in case these disruptions occur. This resembles lower speech-quality ratings by human listeners in such conditions and would reflect the perceptual experience of the listeners which is the aim of our approach. Hence, posteriorgrams that are invariant to noise or distorted speech could not be applied for inferring speech quality. In this study, matched conditions were not considered since the generalization to unseen conditions is especially important in the context of hearing devices.

### 4.2 Inclusion of temporal processing in ASR-based speech quality measures

The comparison of three ASR-based speech quality measures has shown that inverse entropy is a suboptimal measure in the context of speech-specific hearing aid processing although it has provided good results in the past [35, 36]. In contrast to entropy, both M-Measure and MaP explicitly integrate temporal properties of phoneme activations and provide good results for selecting the speech source from competing spatial sources that were identified by an DOA estimator. This hints at the importance of temporal dynamics which are reflected in M-Measure curves for temporal distances of up to 200 ms, which can be attributed to co-articulation effects [13]. The speech-specific properties (that result from using phonemes as DNN training targets) also imply a limitation of our approach since it is not suited to decide which speaker should be attended in a two-speaker setting. This could potentially be resolved by creating speaker-specific DNN models (e.g., by adapting ASR to a speaker) [37-39] or by interpreting eye movements to support users of hearing devices [40-42]. Alternatively, the system could be extended to use a fall-back strategy such as zero-degree enhancement or omni-directional receiver characteristics in multi-speaker scenes.

### 4.3 Temporal integration time and computational requirements

In contrast to earlier work that used observation windows of up to 20 min per condition [17], the temporal integration time in this paper was limited to one utterance, which corresponds to 7.0 s on average with a standard deviation of 2.4 s. Since our approach is tailored to phonetic events that can extend to durations of 300 ms [43], it is probably not suited to provide estimates on a millisecond time scale. However, it should be applicable in acoustic scenes that are stable over a few seconds, and is compatible with methods for speaker tracking in complex acoustic scenes [44].

The DNN used in our model used five hidden layers with 2048 hidden neurons per layer, and estimating the corresponding parameters is computationally demanding, i.e., training should be performed on platforms with graphical processing units (GPUs) for parallel processing. On the other hand, a forward run of the net is relatively cheap and dominated by the multiplications between layers. In a study that analyzed the computational complexity of DNNs and the M-Measure on hearing aid hardware, it was estimated that one forward pass of the DNN and the calculation of the M-Measure can be performed in real time if the number of weights and layers in the DNN are reduced [18]. However, the estimates obtained in [18] are based on a simulation of a typical co-processor, i.e., the corresponding hardware is currently not available. Further, it is not clear if the results presented in the current study can be obtained with the smaller nets that were investigated in [18].

For the full analysis provided in this paper, beamforming and DNN processing was performed for all 19 directions in the frontal hemisphere in parallel, i.e., the computational demand clearly exceeds the resources of hearing aid hardware. For an actual application of the system, it would however be sufficient to compare signal streams based on the dominating sound sources in the acoustic scene, which are identified by the DOA estimator. This should reduce the number of parallel processing streams from 19 to only a few. To circumvent the issue of limited resources on hearing aid hardware, the analysis could be outsourced to connected mobile hardware such as a smartphone for performing the required calculations [45]. This could be a viable strategy since the proposed method does not require a speech synthesis or very low latency.

### 4.4 Combination of ASR-based speech quality measures and DOA information

The results presented in this study indicate that the combination of deep learning, ASR-based speech quality measures, and the probabilistic DOA estimator yield a robust method to identify the location of a speech source using a binaural hearing aid setup. As the method involves steering a spatial filter towards a speech source, it is also applicable for blindly optimizing speech enhancement in hearing aids.

#### 4.4.1 DOA

In this study we have evaluated the localization performance of the proposed system and its two components – the DOA estimation algorithm and ASR-based speech quality measures – with two different noise types in two different environments. In a spatially diffuse noise field, the DOA estimator solves the task of detecting a localized speech source without problems for all SNRs in the test scenario and without noticeable difference between the two environments considered. This result can be explained with the features used in this algorithm, which are based on coherence that is represented by the GCC-PHAT functions used.

In the case of a localized interferer – in this case a vacuum cleaner – the noise source cannot be distinguished from the speech source in terms of coherence. Therefore, the DOA estimator often picks up the noise component instead of the speech signal as the most likely source, even for relatively high SNRs (lower panel in Fig. 6A). This can be attributed to the properties of the vacuum cleaner noise signal, which exhibits a wider spectral bandwidth than speech and dominates the high-frequency bands. Furthermore, the competing speech signal is heavily modulated, i.e., the noise with a comparably small modulation depth dominates the modulation valleys and speech pauses.

#### 4.4.2 ASQMs

The localization performance based on the ASQMs alone shows a largely inconsistent behavior with varying noise type, SNR, and environment. The M-Measure shows its best performance at low SNRs with diffuse noise in the anechoic condition and worst performance in anechoic for all SNRs in case of the localized interferer. Matched Phoneme filters, in contrast, show their best performance in the medium SNR range (around 0 dB) with some trend towards high SNRs in particular for the localized interferer in the reverberant office environment. Inverse entropy is to a large extent unsuitable for speech source localization showing only moderate performance in the least challenging condition (anechoic, diffuse noise, high SNRs). We assume these different observations arise from a complex interaction between the influence of beamforming on the phoneme posteriorgram and the according ASQM: Since the ASQMs are calculated from the same posteriorgrams, the reason for the quite different results for speech localization must origin from the measure itself. However, such an analysis would require an in-depth analysis of the influence of the beamforming on the posteriorgrams and potential interactions with each processing step of each ASQM. Further analysis is therefore beyond the scope of this study, but may be of great interest for future developments. In the context of the current study we have identified an appropriate ASQM for the proposed approach.

#### 4.4.3 DOA × ASQM

By combining the probabilistic DOA estimation method and speech-related spatial information with the DNN and the ASR-based speech quality measures, which, as standalone approaches lack spatial resolution, locating the speech source using the quality measure could be substantially increased in almost all conditions. The differences between the approaches are more pronounced for the case of the localized interferer for which the DOA estimator cannot discriminate between the speech and non-speech source. The combination with the M-measure outperforms the other two approaches except for the case of the localized interferer with low SNRs in the reverberant office environment and the lowest SNR in the anechoic condition. In these cases, the information provided by the DOA estimation algorithm is not sufficiently reliable so that the information from MaP alone turns out to be more robust. However, to switch processing strategies in this particular case, a priori knowledge about SNR and spatial characteristics are required (which is unrealistic for a real-world application), or SNR and spatial parameters need to be estimated, which would introduce additional sources of error, which might mitigate the benefit from switching strategies. It is also noteworthy that, except for the case described above, the performance does not degrade in less complex scenes with diffuse noise only for which the DOA estimator alone provides good results. In summary, the M-Measure is the approach that should be chosen to achieve robust speech source localization. Accordingly, this combination raises the expectation of the optimal performance of the beamforming approach in terms of speech quality.

### 4.5 Future work

Although the presented experiments took into account different room configurations, masker types, and a wide range of signal-to-noise ratios, there are many parameters that need to be studied in future research: So far, two sources in two different rooms were explored, and the problem of detecting the number of sources was not considered. In this work, the observation time for accurate selection of the speech source was investigated. In contrast to our previous study [17], the observation time window was reduced from approximately 20 min to a single utterance. Although the aim of this study was not to investigate the real-time capabilities of the system, the inclusion of instationary scenes (including moving target speakers) is an important research question in this context that needs to be addressed in the future. Further, different noise sources as well as head movements of the listener should be considered. Finally, to make the system more applicable in a hearing aid (which could be supplemented with a smartphone to calculate the DOA estimates and DNN outputs) combining it with an estimator for the number of sources in the scene would be an important step to reduce the computational load caused by the system. Instead of processing the audio signal for all possible directions with the beamformer and computing the phoneme posteriorgrams and quality measure from its output the number of parallel instances can be reduced to a number of candidate direction provided from such an estimator.

### 4.6 Conclusion

This study shows how ASR techniques can be used to improve localization of speech sources and potentially speech enhancement in multi-channel hearing aids without requiring a word decoding. We combined a speech-specific DNN-based measure of speech quality with a probabilistic sound source direction-of-arrival (DOA) estimator. The DOA estimator provides the azimuth-dependent probability of sources, while the DNN output is used to quantify the degradation (or presence) of classified phonemes using three ASR-based speech quality measures (the mean temporal distance, matched filtering, and entropy) with relatively low spatial resolution. We found two of those measures (MM and MaP) to be informative for degradations arising from additive noise, reverberation, and incorrect spatial filtering parameters. They generalize well over these sources of degradation, which is reflected by a high benefit for determining the speech source in the presence of a localized interferer. In diffuse noise conditions, the DOA alone produces excellent results, which are retained in the combined system. A localized interfering source could not be discriminated from the localized speech source based on the information provided by the DOA estimator alone. The combination with the ASR-based speech quality measures could resolve this issue to a large extent.

## Figures and Tables

**Table 1.** Speech source localization performance in hit rate (Eq. (16)) for the diffuse noise scenarios obtained with all different localization approaches – spatial probability only (DOA, Eq. (14)), speech quality measure only (ASQM, Eq. (15)), and the combination of DOA and ASQM (DOA × ASQM, Eq. (13)) – and ASR-based speech quality measures: M-Measure (MM), Matched Phoneme (MaP) filters, and Inverse entropy (iEnt). Results are shown for the anechoic (top panel) and the reverberant office (bottom panel) environment depending on the SNR, the best performing approach per SNR is highlighted in bold.

Anechoic							
SNR	DOA	ASQM			DOA × ASQM		
		MM	MaP	iEnt	MM	MaP	iEnt
−10	**100**	97	13	0	**100**	95	34
−5	**100**	99	45	0	**100**	**100**	69
0	**100**	85	34	0	**100**	**100**	92
5	**100**	57	25	19	**100**	**100**	99
10	**100**	31	27	16	**100**	**100**	**100**
15	**100**	20	17	78	**100**	99	**100**
20	**100**	12	11	44	**100**	98	**100**
Office							
SNR	DOA	ASQM			DOA × ASQM		
		MM	MaP	iEnt	MM	MaP	iEnt
−10	**97**	35	14	0	96	76	3
−5	**100**	35	19	0	**100**	89	27
0	**100**	30	37	0	**100**	93	60
5	**100**	25	38	3	**100**	**100**	95
10	**100**	11	26	6	**100**	97	**100**
15	**100**	7	20	34	**100**	92	**100**
20	**100**	3	6	23	**100**	89	**100**

**Table 2.** Speech source localization performance in hit rate (Eq. (16)) for the scenarios with a vacuum cleaner noise as localized interferer. Obtained with all different localization approaches – spatial probability only (DOA, Eq. (14)), speech quality measure only (ASQM, Eq. (15)), and the combination of DOA and ASQM (DOA × ASQM, Eq. (13)) and ASR-based speech quality measures: M-Measure (MM), Matched Phoneme (MaP) filters, and Inverse entropy (iEnt). Results are shown for the anechoic (top panel) and the reverberant office (bottom panel) environment depending on the SNR, the best performing approach per SNR is highlighted in bold. The SP column denotes the hit rate with respect to the target speech source. In addition, the percentage of estimates that considered the interferer as target is given in the column labeled VC.

Anechoic								
SNR	DOA		ASQM				DOA × ASQM	
	SP	*VC*	MM	MaP	iEnt	MM	MaP	iEnt
−10	0	*100*	1	**60**	3	14	0	0
−5	0	*100*	0	69	1	**86**	2	0
0	0	*100*	0	75	1	**98**	23	22
5	0	*100*	0	66	0	**100**	65	76
10	0	*100*	1	51	1	**100**	91	95
15	7	*93*	3	48	0	**100**	98	99
20	56	*44*	4	49	0	**100**	**100**	**100**
Office								
SNR	DOA		ASQM				DOA × ASQM	
	SP	*VC*	MM	MaP	iEnt	MM	MaP	iEnt
−10	0	*100*	0	**24**	0	0	0	0
−5	0	*100*	7	**50**	1	0	1	0
0	0	*100*	6	**57**	1	24	14	0
5	0	*100*	9	60	0	**77**	35	2
10	0	*100*	6	64	0	**96**	75	69
15	9	*91*	13	79	0	*100*	96	93
20	58	*42*	22	76	0	**100**	**100**	92

## Data Availability

The hearing aid room impulse responses [22] used to generate the audio signals used for beamforming and DOA estimation are available for download from http://medi.uni-oldenburg.de/hrir/. Speech files from the Wall Street Journal Corpus were used, which is commercially available from https://catalog.ldc.upenn.edu/LDC2000T43. The vacuum cleaner noise is commercially available through the BBC Sound Effects Database BBC1991 (Reference VacuumCleaner.BB.ECD3). The computer code used for generating the signals used in this study form the data mentioned above is not publicly available.
